# Biochemical basis of endogenous bioluminescent springtail *Lobella sauteri* (Collembola)

**DOI:** 10.1242/bio.061829

**Published:** 2025-05-12

**Authors:** Manabu Bessho-Uehara, Takumi Kato, Atsuko Ohira, Taizo Nakamori, Yuichi Oba

**Affiliations:** ^1^ The Frontier Research Institute for Interdisciplinary Sciences, Tohoku University, Sendai, 980-8578 Japan; ^2^ Graduate School of Life Sciences, Tohoku University, Sendai, 980-8578 Japan; ^3^ Graduate School of Science, Nagoya University, Nagoya, 464-8602 Japan; ^4^ Tamarokuto Science Center, Tokyo, 188-0014 Japan; ^5^ Faculty of Environment and Information Sciences, Yokohama National University, Yokohama, 240-8501 Japan; ^6^ Department of Environmental Biology, Chubu University, Kasugai, 487-8501 Japan

**Keywords:** ATP, Ecdysis, Laboratory-culture, Bioluminescence, Luciferase, Luciferin

## Abstract

Bioluminescence plays important roles among animals in both intra- and inter-species communication. A variety of bioluminescent organisms inhabit soil environments, even in areas where light penetration is minimal. However, due to the lack of a model system to study underground bioluminescence, the biology and molecular mechanisms underlying this phenomenon remain largely unknown. Springtails (Collembola) are representative soil animals, and we recently identified *Lobella sauteri* (Neanuridae) as a bioluminescent species. *L. sauteri* can be maintained over multiple generations under laboratory conditions on a single food source, the plasmodium *Fuligo septica*, with a generation time of approximately 3 months. Bioluminescence was observed in all developmental stages of *L. sauteri* in laboratory-raised populations. The light emission exhibited periodic changes and increased before ecdysis, coinciding with the whitening of its tubercles. The bioluminescent reaction *in vitro* requires a small molecular (luciferin) fraction, an enzyme (luciferase) fraction, adenosine triphosphate (ATP), and Mg^2+^. Comparative transcriptomic and biochemical analyses suggest that *L. sauteri* employs a novel endogenous bioluminescent molecular mechanism. We propose that *L. sauteri* provides a valuable research opportunity for investigating novel bioluminescence systems and underground light-based communication.

## Teaser

**Summary:** A novel ATP-dependent bioluminescent system discovered in a tiny insect that can be reared in a desk drawer.

## INTRODUCTION

Bioluminescence is a visible light-producing phenomenon caused by a chemical reaction in living organisms (Harvey, 1952; Shimomura and Yampolsky, 2019). The light emission reaction involves small organic compounds as substrates and enzymes, generally called luciferin and luciferase, respectively, along with oxygen, with or without cofactors. In some cases, luciferin and luciferase exist as a stable complex, which is specifically referred to as a photoprotein. The structures of luciferins and the amino acid sequences of luciferases are identical and homologous, respectively, within lineages that share an evolutionary origin of bioluminescence (Fallon et al., 2018; He et al., 2024; Lau et al., 2025; Schnitzler et al., 2012). The evolutionary origins of bioluminescence were estimated more than 100 times but the majority of the chemical molecules and genetic bases remain unknown (Hastings, 1995; Davis et al., 2016; Lau and Oakley, 2021; Bessho-Uehara et al., 2024). The discovery of a novel bioluminescent system expands its applications in life sciences and related disciplines.

Bioluminescence has been reported in more than 900 genera across a variety of living organisms, ranging from prokaryotic bacteria to multicellular organisms, including mushrooms, fungus gnats, fireflies, click beetles, millipedes, earthworms, and many other terrestrial and marine organisms (Oba et al., 2011; Oba, 2022; Claes et al., 2024; Duchatelet and Dupont, 2024; Schramm and Weiß, 2024). The luminescent mechanisms of mushrooms and beetles have been well studied from a chemical perspective. While both mushrooms and beetles emit green light, the biochemical mechanisms underlying their luminescence are distinct, reflecting their separate evolutionary pathways and chemical foundations (Fallon et al., 2018; Kotlobay et al., 2018).

Bioluminescent organisms can be categorized into two groups: those that produce their own luminescent compounds (autogenic bioluminescence) and those that rely on external sources (non-autogenic bioluminescence). Both mushrooms and beetles are known as autogenic bioluminescence and can synthesize their own luminous compounds, such as luciferin, luciferase, and necessary cofactors (Fallon et al., 2018; Kotlobay et al., 2018). Non-autogenic bioluminescence can be further classified into two distinct categories: (1) organisms that acquire luminous factors (e.g. luciferin and, in rare cases, luciferase) through their diet, and (2) organisms that depend on symbiotic relationships with bioluminescent bacteria (Haygood, 1993; Nealson and Hastings, 1979; Tanet et al., 2020). Several organisms require dietary acquisition of luciferin to sustain their bioluminescence. Some species have been experimentally confirmed to rely on dietary luciferin acquisition: *Neognathophausia ingens*, *Aequorea victoria*, *Eutonia indicans, Amphiura filiformis*, which all acquire coelenterazine, and *Porichthys notatus*, which obtains vargulin through its diet (Coubris et al., 2024; Frank et al., 1984; Haddock et al., 2001; Warner and Case, 1980). An extreme case is observed in the luminous fish *Parapriacanthus ransonneti*, which acquires both luciferin (vargulin) and luciferase through its diet (Bessho-Uehara et al., 2020; Johnson et al., 1961). On the other hand, many organisms rely on symbiotic relationships with bioluminescent bacteria. The Hawaiian bobtail squid, *Euprymna scolopes*, which harbors *Aliivibrio fischeri* (formerly *Vibrio fischeri*) in specialized symbiotic organs, many other species exhibit similar dependencies (Nealson and Hastings, 1979; Nyholm and McFall-Ngai, 2004). Numerous fish species, including flashlight fish (Anomalopidae), anglerfish (Ceratioidei), and ponyfish (Leiognathidae), as well as certain squid species, use symbiotic bioluminescence for communication, camouflage, and predation. Reviews such as Nealson and Hastings (1979), Haygood (1993), Tanet et al. (2020), and Duchatelet and Dupont (2024) provide a broader overview of bacterial bioluminescence, and its ecological significance. The bioluminescence mechanisms of many luminous organisms, such as soil-dwelling animals, remain largely unstudied. Further research is necessary to understand the evolutionary and ecological roles of bioluminescence in these lesser-known taxa.

The order Collembola (springtails) are members of the hexapods, which share a common ancestor with insects and diverged approximately 400 million years ago (Misof et al., 2014). Bioluminescence in springtails is one of the least studied phenomena among those in hexapods, despite having been reported as early as 1851 (Allman, 1851). It is not known whether luminous springtails are autogenic or non-autogenic bioluminescence. Springtails typically live in the litter layer that contains the luminous fungi. The midgut of the luminous springtail species *Neanura muscorum* (family Neanuridae) was found to be full of luminous mycelia but species from other localities were nonluminous, suggesting that the light originates from fungi (Harvey, 1952). Sano et al., (2019) suggested that luminescence of *Lobella* sp. (Neanuridae) is independent from bacterial or fungal symbiosis because of the bioluminescent behavior of *Lobella* sp. was oscillatory responding to an external factor, and also histological observation suggested that eosin-positive granules in the light source, mainly in the tubercles on the thorax and abdomen, are present in the luminous specimen but not in the closely related non-luminous specimen (Sano et al., 2019). The chemical study on bioluminescence using identified species has not been accomplished due to the difficulty of identifying species and the taxonomic confusion (Oba et al., 2011; Sano et al., 2019). This has posed challenges for reproducibility in research. Previously, we conducted a taxonomic review of the genus *Lobella* (family Neanuridae) and found that *Lobella sauteri* (Börner, 1906), *Lobella yambaru* (Tanaka and Hasegawa, 2010), *Vitronura giselae* (Gisin, 1950), and *Vitronura kunigamiensis* Tanaka and Hasegawa, 2010 are bioluminescent (Ohira et al., 2023).

Establishment of laboratory culture is an essential advancement to study various aspects of biology including behavior, development, genetics, and biochemistry. In this study, we successfully cultured *L. sauteri* under laboratory conditions, allowing us to investigate the biochemical basis of its bioluminescence. We found that *L. sauteri* can emit light throughout its life cycle. The bioluminescence of *L. sauteri* involves luciferin, luciferase, ATP, and Mg^2+^, with both luciferin and luciferase being endogenously produced independent of dietary supplementation. Additionally, we observed periodic increases in bioluminescence synchronized with changes in tubercular color.

## RESULTS AND DISCUSSION

### Laboratory culture of *L. sauteri*

**Fig. 1.**
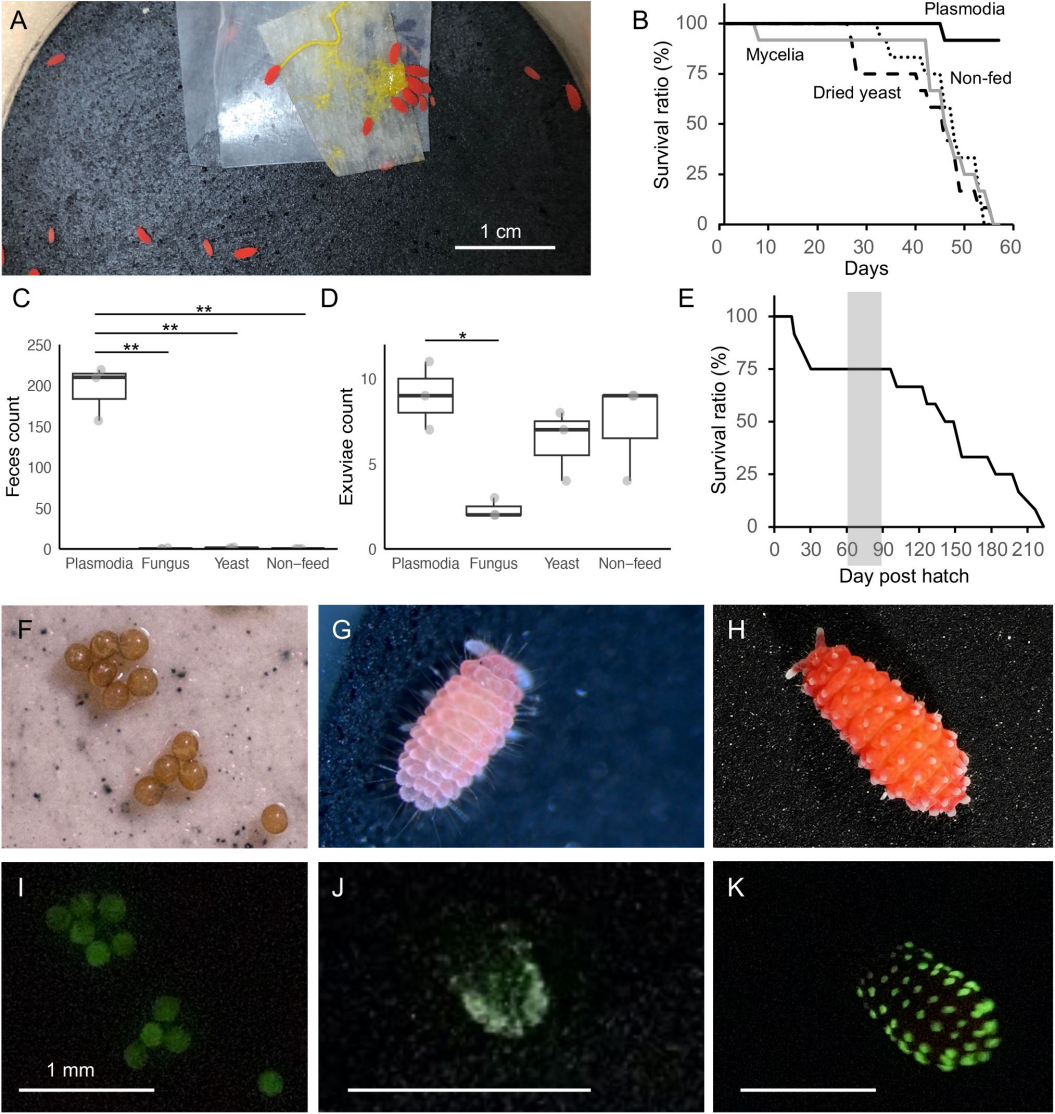
**Culture of *Lobella sauteri* and its innate bioluminescence.** (A) Laboratory setup of the culture system for *L. sauteri.* The specimens were kept in a plastic container embedded with plaster. A piece of paper with plasmodia was placed on the plaster once or twice a week. (B) Survival curves of wild-caught *L. sauteri* in the four culture conditions. The specimens cannot live longer than about 2 months under non-feeding conditions (dotted line) or when fed with mycelia (gray line) or dried yeast (dashed line) but survived when fed plasmodia (black line). (C,D) Feces (C) and exuviae (D) produced by four individuals in a week were compared among different feeding conditions. Each value represents the mean of three independent biological replicates, presented as dots on the bar. *P* values of post-ANOVA Tukey HSD test are indicated by single (*) and double asterisks (**) for *P*-value<0.05 and *P*-value<0.01, respectively. (E) Survival curve of the newly hatched specimens (*n*=12) raised under our culture conditions. Eggs were observed between 60-90 days after hatching (gray shaded period). (F-K) Bioluminescence of eggs, juvenile, and adult of laboratory-cultured specimens. Photographed for 10 s, F/2.8, ISO 51,200 for eggs; and 5 s, F/2.8, ISO 51,200 for an adult individual.

We successfully raised *L. sauteri* under laboratory conditions (Fig. 1A). *L. sauteri* was fed with the plasmodia *Fuligo septica* at 22°C for a long period (Fig. 1B). Wild-caught *L. sauteri* specimens that were not fed survived as long as those fed on *Mycena chlorophos* mycelium and dry yeast, suggesting that *L. sauteri* did not feed on these substrates (Fig. 1B). Survival analysis using the log-rank test revealed a significant overall difference among the four feeding conditions [χ²(3)=30.0, *P*<0.01]. *Post hoc* pairwise comparisons with Bonferroni correction showed that a significant increase in survival was observed only in the plasmodia-fed group (Fig. 1B, *P*<0.01 for comparisons between plasmodia-fed group and all other groups; *P*>0.05 for comparisons among the non-feeding, yeast-fed, and fungus-fed groups). The activity of the springtails was measured by counting the number of feces and exuviae observed over 4 weeks, from the third to the sixth week, showing significant differences in both indicators. The number of feces was significantly greater in the plasmodia-fed condition compared to the other conditions (Fig. 1C, ANOVA, *F*_3, 8_=101, *P*<0.01; post-ANOVA Tukey's honestly significant difference (Tukey's HSD) test, *P*-value<0.01). The number of exuviae showed no significant differences between the non-feeding condition and the other conditions (Fig. 1D, *F*_3, 8_=5.67, *P*<0.05), while it was significantly lower in the fungus-fed condition than in the plasmodia-fed condition (post-ANOVA Tukey's HSD test, *P*-value=0.018). The mature specimens laid egg masses consisting of approximately 10±1.0 eggs, and 86% of them hatched in an average of 15.5±1.9 days (*n*=26) (Table S1). The hatched juveniles were reared under conditions where plasmodia served as their food source. The eggs were observed 60-90 days after hatching (gray ribbon in Fig. 1E). The mortality rate was high during the first month post-hatching, primarily due to the entrapment of young, small-sized individuals in water droplets within the rearing containers. Subsequently, they exhibited stable growth, with gradual mortality occurring between 90 and 220 days, likely due to senescence. The median lifespan was 140 days, and 154 days when excluding individuals that died due to early entrapment in water droplets. The combined duration from egg-laying to hatching and from hatching to the next generation was approximately 75 to 105 days under our laboratory rearing conditions.

The establishment of a laboratory culture of luminous Collembola is important for studying bioluminescence with reproducibility. Using specimens with reliable taxonomic identification can provide reproducible results for chemistry, ecology, and evolutionary research. The culture of other Neanuridae springtails is reported, including the luminous species *Neanura muscorum* and non-luminous species (Hoskins et al., 2015; Kataoka and Nakamori, 2020). The life cycle of *L. sauteri,* which is about 3 months in our culture conditions and similar to that of *N. muscorum,* is realistic and endurable for applying genetic approaches. Comparison with related species will help our understanding of the biology of luminous Collembola.

### Endogenous bioluminescence in *L. sauteri*

The light-emitting capacity of *L. sauteri* was observed throughout its life cycle. Eggs emitted the continuous dim green light spontaneously from the whole body (Fig. 1F,I). Juvenile and adult individuals of *L. sauteri* emitted green light from the dorsal tubercles when mechanically stimulated by a paintbrush or when blown on (Fig. 1G,H,J,K). The fifth generation in the laboratory raised with the plasmodia as a single food source maintained the bioluminescent ability.

Dietary acquisition of the luminous materials, such as luciferin and luciferase, is known from many luminous marine organisms (Bessho-Uehara et al., 2020; Haddock et al., 2001). The involvement of luminous fungi was once proposed since the midgut of the luminous springtail *N. muscorum* was found to be filled with luminous mycelia (Harvey, 1952). However, behavioral and histological observations suggested endogenous luminescence in *Lobella* sp., which emits light from the tubercles (Sano et al., 2019). The luminosity maintained in our laboratory culture for five generations ruled out the requirement of the dietary supplementation of luminous materials from its natural diet. The possibility that the non-luminous plasmodia *F. septica* provides luminescent factors to *L. sauteri* is unlikely, which is discussed later. These results suggest that *L. sauteri* has an endogenous bioluminescent system.

### *In vitro* reconstruction of bioluminescence

**Fig. 2.**
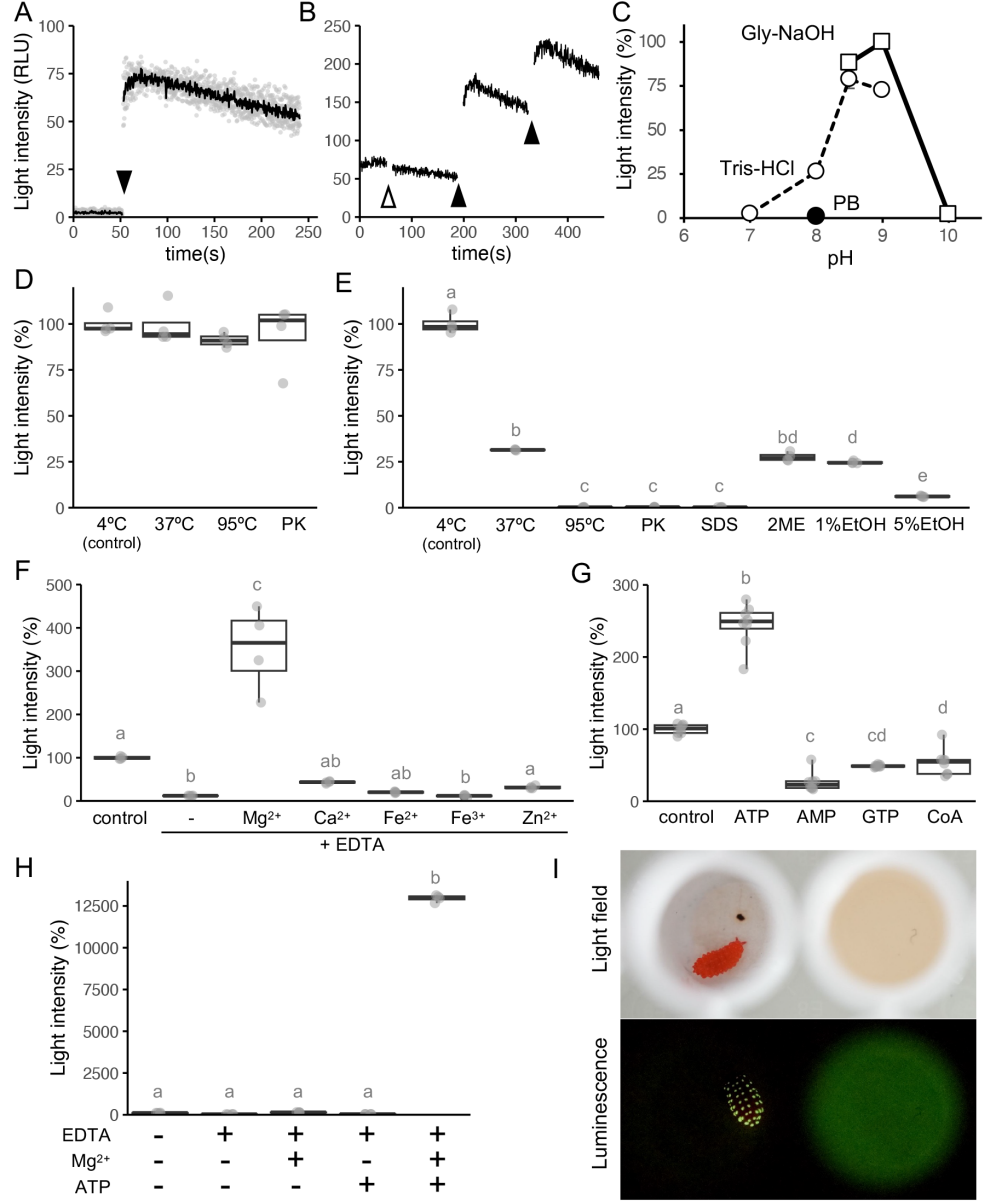
**Biochemical characterization of *Lobella sauteri* bioluminescence.** (A) Time course of luminescence reaction initiated by the addition of the small molecular (containing luciferin) fraction (filled triangle) to the large molecular (containing luciferase) fraction. The black curve represents the mean of replicates (*n*=3). (B) Time course of luminescence reaction initiated by the addition of luciferase fraction (open triangle) or luciferin fraction (filled triangle). (C) Effect of pH on luminescence activity with buffers of Tris-HCl (open circle), glycine-NaOH buffer (open square), or sodium phosphate buffer (PB, closed circle). (D) Effect of heat (4°C, 37°C, and 95°C) and protease treatment (PK) on luciferin activity. (E) Effect of heat (4°C, 37°C, and 95°C), protease (PK), detergent (SDS), reductant (2ME), and organic solvent (1% or 5% EtOH) on luciferase activity. (F) Effect of cations in the presence of EDTA on luciferase activity. (G) Effect of ATP, AMP, GTP, and CoA on luciferase activity. (H) Effect of combination of magnesium ion and ATP on the luminescence reaction in glycine-NaOH buffer at pH 9.0. (I) Long-time exposure of *in vivo* and *in vitro* luminescence of *L. sauteri.* The individual and the reaction mix are placed in the white 96-well plate and photographed with exposure time 5 s, F/2.8, ISO 51,200.

We extracted and separated active materials by ultracentrifugation to reconstruct the bioluminescent reaction. The light intensity increased after adding the small molecular fraction extract to the large molecular fraction extract (Fig. 2A). For clarity, we refer to the low molecular weight fraction as the luciferin fraction and the high molecular weight fraction as the luciferase fraction hereafter. The light intensity (approximately 100 in the relative light unit, RLU) was significantly higher than the background. However, although the bioluminescent components in the reaction mixture were intended to represent one-third of whole body of an adult individual, the resulting light intensity was much weaker than that of a single living specimen (spontaneous flashes can exceed 10,000 RLU). Repeated addition of the luciferin fraction into the reaction mixture increased light intensity, suggesting that the fraction contains luciferin (Fig. 2B). The photoprotein system is biochemically defined by the fact that the total light production is proportional to the amount of photoprotein but not to the amount of luciferin (Shimomura and Yampolsky, 2019). Our results suggest that the bioluminescence system of *L. sauteri* is a luciferin–luciferase system rather than a photoprotein system.

To investigate the optimum pH condition for the *in vitro* bioluminescence reaction of *L. sauteri,* we tested the reaction in several pH buffers. The reaction showed the highest activity at pH 9.0 in glycine-NaOH buffer, while almost no activity was observed at pH 7.0 in Tris-HCl buffer and pH 10.0 in glycine-NaOH buffer (Fig. 2C). Notably, the reaction did not produce light when sodium phosphate buffer at pH 8.0 was used. This result implies that the reaction was inhibited by phosphate, as in the cases of other biochemical reactions involving ATP and magnesium ions (Mg^2+^) such as firefly luciferase and ATPase (Robinson et al., 1978; Webster et al., 1980).

Generally, luciferins are heat-tolerant small molecules, while luciferases are sensitive to temperature and other protein-denaturing conditions. Luciferin and luciferase of *L. sauteri* were tested and followed this trend. Luciferin maintained its luminescent activity when incubated at 37°C or 95°C for 5 min or treated with protease (Fig. 2D, *F*_3,12_=0.59, *P*=0.63). On the other hand, luciferase lost 69% and 99% of its activity after incubation for 30 min at 37°C or 95°C, respectively (Fig. 2E). The loss of activity after protease treatment supports the hypothesis that the light-producing activity in the large molecular fraction is due to luciferase as an enzymatic protein, rather than the alternative hypothesis that the catalyst is a ribozyme or other material (Fig. 2E). The stability of luciferase was also tested with several denaturing conditions (Fig. 2E, *F*_7,23_=957, *P*<0.01). The tolerance to reduction suggests that disulfide bonds are less essential for luciferase activity. Luciferase is sensitive to sodium dodecyl sulfate (SDS) and ethanol, suggesting that detergents and organic solvents should be avoided to extract luciferase or assay luminescence activity.

We further investigated possible cofactors involved in the luminescent reaction of *L. sauteri* and found that Mg^2+^ was required for the reaction. The luminescent reaction was inhibited in the presence of EDTA, which chelates divalent cations (Fig. 2F, *F*_6,21_=44.3, *P*<0.01). Then, we screened several cations (Mg^2+^, Ca^2+^, Fe^2+^, Fe^3+^, and Zn^2+^) and found that Mg^2+^ recovered and enhanced the luminescent reaction (Fig. 2F).

We also investigated other cofactors using reagents (ATP, AMP, GTP, and CoA) available in the laboratory and found that ATP is involved in the luminescent reaction (Fig. 2G, *F*_6,21_=44.3, *P*<0.01). The involvement of ATP in the luminescent reaction is supported by the fact that it was lost in the presence of phosphate (Fig. 2B).

Taken together, we found that small molecular substrate (luciferin), enzymatic protein (luciferase), ATP and Mg^2+^ are essential to reconstruct the *in vitro* bioluminescent reaction of *L. sauteri.* Luciferin and luciferase can be extracted by aqueous buffer and separated by size fractionation using ultracentrifugation. The light producing reaction can be reconstructed by mixing luciferin fraction and luciferase fraction in the presence of Mg^2+^ and ATP at basic buffer conditions such as glycine-NaOH buffer at pH 9.0. Using this condition, the light intensity increased more than 100 times compared to the homogenate alone at pH 9.0 (Fig. 2H, *F*_4,15_=16,151, *P*<0.01). The *in vitro* luminescence produced by this reaction condition was similar to dim green light of a live individual (Fig. 2I). *In vitro* luminescence was observed using long-exposure photography with a high-sensitivity digital camera. The faint light produced was similar to that of living springtails in nature, which is barely visible to dark-adapted naked eyes. Therefore, we could not determine the *in vitro* luminescence spectrum due to the low light intensity and limited material availability.

The involvement of ATP and Mg^2+^ in the bioluminescence reaction has been reported in beetles (click beetle, firefly, and its relatives), Australian dipterans *Arachnocampa richardsae*, Siberian potworms *Fridericia heliota*, firefly squids *Watasenia scintillans*, and millipedes *Motyxia sequoiae* (formerly *Luminodesmus sequoiae*) (Fallon et al., 2018; Lee, 1976; Petushkov et al., 2014; Shimomura, 1981; Shimomura and Yampolsky, 2019; Tsuji, 1985)*.* The former four systems are the luciferin–luciferase system, while the photoprotein system is reported in the millipedes. The chemical structures of luciferins in these systems are not identical. Although genes similar to beetle luciferase have been reported in dipterans and squids, luciferase activity of the recombinant protein has been characterized and demonstrated only in beetles (Gimenez et al., 2016; Watkins et al., 2018; Yoshida et al., 2020). Fireflies are the only luminous organisms geographically overlapping with the luminous springtail *L. sauteri* (Oba et al., 2011; Ohira et al., 2023; Osozawa et al., 2015). Springtails inhabit a wide range of environments, from water edges to terrestrial habitats, and exhibit diverse feeding habits, including the consumption of fungi, bacteria, plant-derived materials, and microfauna (Hopkin, 1997). Notably, members of the family Neanuridae, to which the bioluminescent species *L. sauteri* belongs, are suggested to occupy relatively high trophic levels based on nitrogen stable isotope analysis (Chahartaghi et al., 2005; Potapov et al., 2016) and are known to consume animal-derived organic matter, including the carcasses of crustaceans, gastropods and earthworms (Joose, 1966; An et al., 2013). The trophic link is not known between fireflies and springtail, but it is unlikely that the luciferin supplement of *L. sauteri* depends on fireflies through the food web because both can maintain bioluminescence capability in the laboratory culture condition for generations (Fig. 1) (Fallon et al., 2018). Considering the luciferin diversity among those animals, it is possible that the springtail *L. sauteri* has a novel luciferin, despite the common requirement for Mg^2+^ and ATP.

### Novel bioluminescent system of *L. sauteri*

We further investigated the genes involved in the bioluminescent reaction in *L. sauteri* by transcriptome analysis. We obtained high-quality total RNA with RNA integrity number (RIN) of 9.4 from a single individual of *L. sauteri.* The short-read, next-generation sequencing using NovaSeq yielded 151 bp paired-end raw reads totaling 10 Gbp. The *de novo* transcriptome assembly was used for gene prediction using TransDecoder software, yielding 19,387 coding sequences (CDS) with a contig N50 of 1758 bp. The amino acid sequences were predicted from the CDS using TransDecoder. The benchmarking universal single-copy orthologs (BUSCO) score for Arthropoda (arthoropoda_odb12) was [complete: 78.9% (complete and single-copy: 21.0%, complete and duplicated: 57.9%), fragmented: 4.9%, missing: 16.2%, *n*: 1667] suggested that our predicted protein model recovered majority of the proteins in *L. sauteri*.

We found no single-copy ortholog genes to the known luciferases. The BLAST search using known luciferase protein sequences from 16 groups, including both terrestrial and marine luminous organisms, against the *de novo* transcriptome dataset of *L. sauteri* detected proteins similar to luciferases of fireflies (*Photinus pyralis* luciferase, P08659.1), click beetles (*Pyrophorus plagiophthalmus* luciferase, AAQ11735.1), and a luminous squid (*Sthenoteuthis oualaniensis* symplectin, C6KYS2.2), with e-values lower than 1e-10 and bit scores greater than 100.

However, these BLAST search hits showed higher similarity to the other annotated genes. For example, in the case of the BLAST search results for symplectin, a photoprotein of luminous squid, we found the predicted protein LobSau_DN6472_c0_g2_i4.p1 with the e-value of 1e-31, but this protein showed higher similarity to the pantetheinase isoform X2 in the non-luminous springtail *Folsomia candida* with the e-value of 5e-103. The cysteine at position 390 in symplectin, where its luciferin, dehydrocoelenterazine, binds, is essential for its light-emitting function (Isobe et al., 2008), but it was not conserved in the predicted protein LobSau_DN6472_c0_g2_i4.p1. Symplectin is a photoprotein that emits blue light with a peak at 456 nm in the presence of monovalent cations, but Mg^2+^ does not cause light emission (Tsuji and Leisman, 1981, pp. 6179–6723). Thus, it is unlikely that the symplectin homolog (LobSau_DN6472_c0_g2_i4.p1) is a luciferase of *L. sauteri.*

**Fig. 3.**
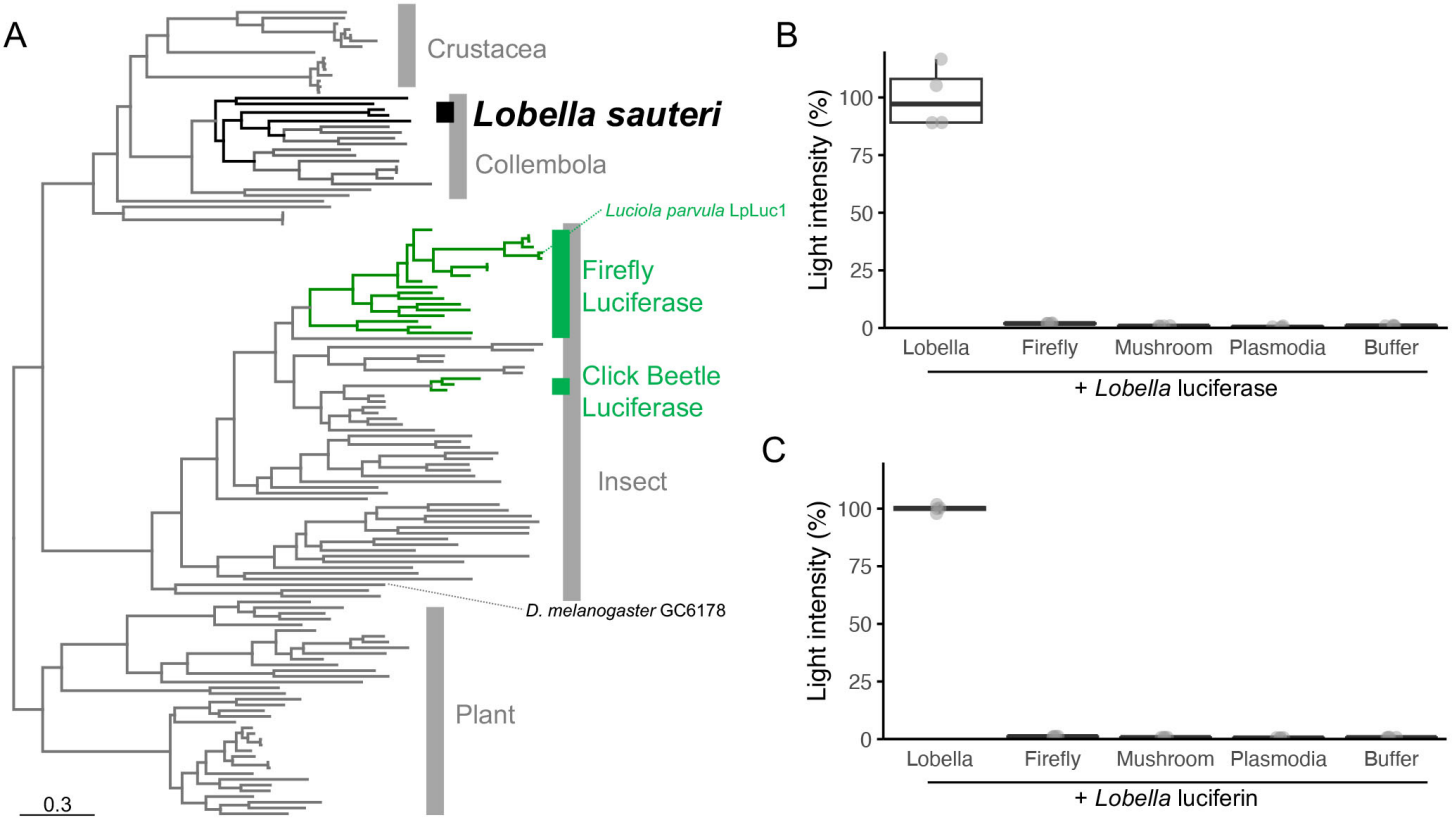
**Phylogeny of beetle luciferase homologs in *Lobella sauteri* and cross-reactivity of bioluminescent reaction with firefly, mushroom and plasmodia.** (A) Maximum Likelihood tree of the acyl CoA ligase family including firefly and click beetle luciferases (green). Grey OTUs indicate homologs but not luciferase (represented GC6178 of *Drosophila melanogaster*). The predicted proteins of *L. sauteri* in our RNA-seq analysis were located in the separate clade in Collembola (black). (B) Luciferin activity of small molecular fractions from *L. sauteri,* luminous mushroom *M. chlorophos* and non-luminous plasmodia *F. septica,* and authentic firefly luciferin. (C) Luciferase activity of large molecular fractions from *L. sauteri, M. chlorophos, F. septica,* and purified recombinant luciferase of firefly *Luciola parvula* (indicated as LpLuc1 in panel A)*.*

For the homologs to the luciferases of fireflies and click beetles, we found four predicted proteins (sequence IDs: LobSau_DN126_c0_g1_i1.p1, LobSau_DN305_c0_g1_i3.p1, LobSau_DN5132_c0_g1_i1.p1, and LobSau_DN12441_c0_g2_i5.p1) with e-values between 5e-84 and 5e-91. In the phylogenetic tree, the four detected proteins of *L. sauteri* formed a clade with the 4-coumarate-CoA ligase of the non-luminous collembola *F. candida*, which is distantly related to the beetle luciferases. Firefly and click beetle luciferases formed monophyletic clades, respectively (Fig. 3A). The firefly lineage and the click beetle lineage evolved bioluminescence in parallel, with both of their luciferases evolving from the fatty acyl CoA ligase family and requiring Mg^2+^ and ATP (Fallon et al., 2018; Oba et al., 2009). The amino acid sequences of those genes of *L. sauteri* showed higher similarity to the non-luciferase proteins in beetles than to beetle luciferases. The AMP-binding sites are conserved in those proteins, as well as in other CoA ligases. On the other hand, five or six out of seven key amino acid residues for the possible interaction with beetle luciferin were not conserved (Fig. S1) (Nakatsu et al., 2006). To test the possibility that *L. sauteri* uses beetle luciferase homologs and beetle luciferin in its bioluminescent reaction, we tested cross-reactions between extracts of *L. sauteri* and the firefly bioluminescent system. The luciferase extract of *L. sauteri* did not produce light when reacted with firefly luciferin (Fig. 3B). The luciferin extract of *L. sauteri* did not produce light when reacted with a purified recombinant luciferase of the firefly *Luciola parvula* (Fig. 3C)*.* The involvement of Mg^2+^ and ATP in the *L. sauteri* luminescence reaction and the geographical overlap between *L. sauteri* and fireflies suggest a potential chemical trophic link between them. However, both luciferin and luciferase were shown to be unrelated to those of firefly bioluminescent systems. It is reasonable to propose that a different bioluminescent mechanism exists in Collembola compared to luminous beetles, considering that bioluminescence in beetles is estimated to have emerged about 140 million years ago, whereas the Collembola lineage diverged from the beetle lineage approximately 400 million years ago (Fallon et al., 2018; Misof et al., 2014; Powell et al., 2022).

### Endogenous bioluminescence of *L. sauteri*

We further tested the possibility of dietary supplementation of the luminous materials for Collembola bioluminescence. We tested cross-reactions using the extracts of plasmodia. We also tested the cross-reactions using hispidin, the luciferin precursor of luminous mushrooms, and the extract of the luminous mushroom *M. chlorophos,* because the involvement of the luminous mushroom in Collembola bioluminescence had been suspected (Harvey, 1952, p. 649). The luciferase of *L. sauteri* did not produce light when reacted with luciferin extracts from the luminous mushroom *M. chlorophos* or plasmodia *F. septica* (Fig. 3B)*.* The luciferin of *L. sauteri* did not produce light when reacted with luciferase extracts from the luminous mushroom *M. chlorophos* or plasmodia *F. septica* (Fig. 3C)*.* We believe that the negative result is not due to the low amounts of luciferin or luciferase because the raw materials used were ten times greater in wet weight compared to *L. sauteri*, and an excess amount of authentic luciferin and purified luciferase, LpLuc1, were used. The plasmodia *F. septica* have been used as a single food source in our culture system, which successfully raised the fifth generation while maintaining bioluminescence. The negative results of the cross-reaction suggest that plasmodia neither supply luciferin nor luciferase. Thus, *L. sauteri* can produce its luciferin and luciferase by itself. The symbiosis or involvement of luminous fungus has been discussed before, but the results of biochemical tests showed the independence of the luminous mechanism in *L. sauteri* from that of mushrooms. Our results support the self-luminescence suggested in *Lobella* sp. from Japan through behavior and histological observation (Sano et al., 2019).

Combining the results of observation, bioinformatics analyses and biochemistry experiments, it is suggested that the luminous springtail *L. sauteri* possesses a novel molecular mechanism for its endogenous bioluminescence, distinct from other known bioluminescent systems. Revealing the molecules and genes involved in the bioluminescent system in *L. sauteri* gives an insight that may lead to future applications in bioengineering and life sciences.

### Enhanced bioluminescence before ecdysis

**Fig. 4.**
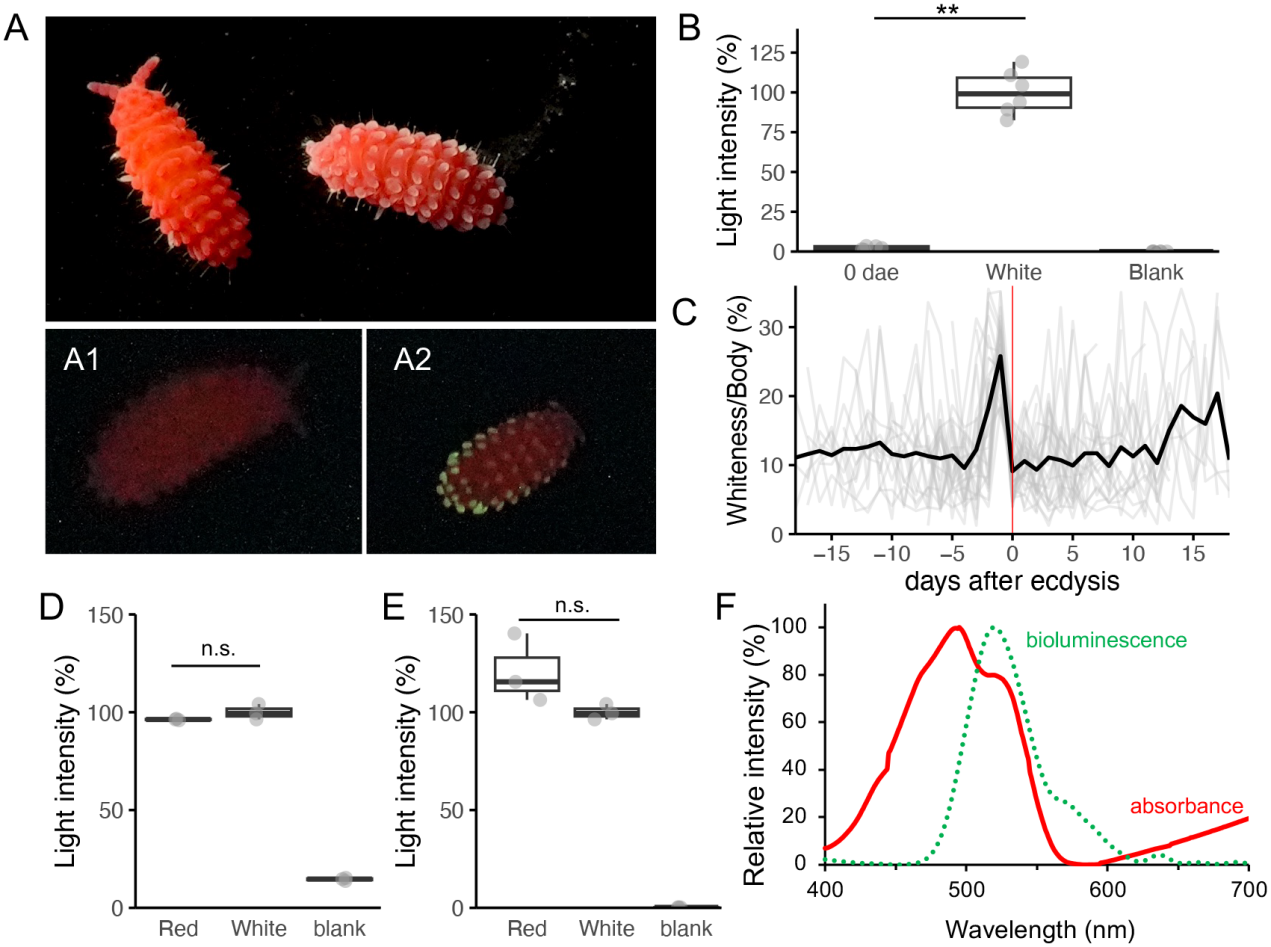
**The relationship between the tubercle colors, ecdysis, and bioluminescence.** (A) Photographs for tubercle coloration under white light with an exposure time of 1/200 s, F/8.0, ISO 52,000 (top) and for the bioluminescence of red (A1) and white (A2) tubercle specimens in the dark with an exposure time of 1/20 s, F/2.8, ISO 204,800 (bottom). (B) Total luminescence capacity of red (0 days after ecdysis, dae) and white tubercule individuals. (C) Changes in the white area over the ecdysis cycle. The dates of the observed exuviae were adjusted to day zero (red vertical line). The mean value (black curve) is calculated from data of 20 individuals (gray curves). Note that if ecdysis events were observed more than twice during the experimental period (19 days), the curve data were used multiple times for each ecdysis event. (D,E) Biochemical detection of luciferin (D) and luciferase (E) from red (0 days after ecdysis) and white tubercule individuals. (F) The absorbance spectrum of crude red pigment extract (red) overlaid on the bioluminescence spectrum (from Ohira et al., 2023). Significant differences in light intensities between samples are indicated by double asterisks (**) or n.s. for *P*-value<0.01 and no significance (*P*-value>0.05), respectively (Student's *t*-test).

During daily observation of the specimens, we noticed that some individuals emitted stronger light than others, and it was correlated with the color of their tubercles. Live *L. sauteri* specimens with white tubercles emitted brighter light than specimens with red tubercles when mechanically stimulated (Fig. 4A) (Movie 1). The total bioluminescence capacity of each specimen was measured by thawing frozen specimens to ensure the full consumption of luminous substrate, minimizing the variability caused by the blinking nature of natural bioluminescence in *L. sauteri*. The total light intensity of specimens with white tubercles was greater than that of specimens with red tubercles (Fig. 4B, Student's *t*-test, t_9_=−15.5, *P*<0.01). The changes in tubercle pigmentation were monitored individually. An analysis of the white areas in the photographed dorsal images revealed that the color of the tubercles changed drastically from red to white 1 or 2 days before ecdysis (Fig. 4C). Under laboratory conditions, when fed abundant *F. septica* plasmodia at 22°C, *L. sauteri* molts every 12.8±3.9 days on average (*n*=1600), and the tubercles turn red again after ecdysis. This color change cycle associated with ecdysis occurs throughout the life of *L. sauteri*, although it may not be apparent in small young individuals whose red pigmentation has not yet fully developed.

We determined the relative levels of luciferin and luciferase activities to assess whether they correspond to *in vivo* bioluminescence and total light emission from frozen specimens. The luciferin and luciferase fractions extracted from white and red tubercle specimens were mixed in all combinations to measure relative activity. The relative activity of luciferin did not significantly change between white and red tubercular specimens (Fig. 4D, t_4_=−1.62, *P*=0.18). The relative amount of luciferase also did not significantly change between white and red tubercular specimens (Fig. 4E, t_4_=1.99, *P*=0.12). These results suggest that the quantity of luminescent materials, specifically luciferin and luciferase, in the whole body of the specimens does not explain the changes in natural bioluminescence related to ecdysis.

The red pigment extracted from *L. sauteri* showed spectral absorbance with peaks at 499 nm and 520 nm (Fig. 4F). The latter absorbance peak corresponds to the bioluminescence peak at 520 nm (Ohira et al., 2023). The correspondence of the peak absorption and bioluminescence gives insight that changes in red pigment development regulate bioluminescence in *L. sauteri*. Identifying the red pigment and its localization is necessary to understand bioluminescence regulation in *L. sauteri*. Due to material limitations, this was not pursued in this study.

The tubercular luminescence in *L. sauteri* intensified before ecdysis, but the amount of luciferin and luciferase did not chang significantly (Fig. 4A-E). The intensity of *in vivo* bioluminescence might be regulated by red pigmentation on the tubercles. This indicates that *L. sauteri* regulates bioluminescence in two independent modes: the days-order long-term regulation by the development of the red pigmentation*,* and the seconds-order short-term regulation, likely under neural or hormonal control (Ohira et al., 2023; Sano et al., 2019). Long-term regulation might be controlled by hormones (e.g. ecdysone or juvenile hormone) that modify the tubercle morphology, but experimental verification is required to confirm this hypothesis. Short-term regulation, on the other hand, might be controlled by the nervous system based on the following observations: live specimens do not emit light spontaneously and continuously but emit light in response to stimulation within seconds. This rapid regulation of bioluminescence is known to occur through neural control, as investigated in fireflies and certain fish species, or through mechanical control, as seen in ponyfish and flashlight fish, which regulate light emission by opening or closing a shutter to conceal or expose continuous bacterial bioluminescence (Johnson and Rosenblatt, 1988; Haygood, 1993; Trimmer et al., 2001). However, the involvement of hormones, which can act via second-order reactions – as is the case with adrenaline – remains a possibility. Further physiological experiments are required to elucidate the regulatory mechanisms of bioluminescence in *L. sauteri.*

The ecological role of enhanced luminescence before ecdysis has not been discussed, to our knowledge. Enhanced bioluminescence after ecdysis is reported in some firefly larvae, possibly to enhance the aposematism effect, signaling their toxicity when the cuticle is not hardened, although this has not been experimentally tested (Bessho-Uehara et al., 2017; Viviani et al., 2008). The possible roles of enhanced bioluminescence before ecdysis might include (a) aposematism during the vulnerable period before ecdysis, though it is unclear if *L. sauteri* has any defense mechanisms for aposematism and is vulnerable before ecdysis, and (b) a mating signal timed with ecdysis, though its importance for reproduction is unknown. The specific natural enemies of *L. sauteri* remain unknown. However, common predators of springtails include mites, ants, beetles and frogs. In the case of another bioluminescent springtail species, *Neanura muscorum*, it has been observed that predators tend to avoid them, and they are known to possess deterrents (Messer et al., 2000). Similarly, *L. sauteri* has been observed to secrete defensive fluids in response to stimuli (Ohira et al., 2023), suggesting that it may also be avoided by predators. Further research is needed to clarify this potential relationship. The stable culture system of *L. sauteri* that we developed in this study provides an opportunity to study the ecological aspects of bioluminescence.

### Conclusion

Bioluminescence in Collembola is one of the least studied groups of animals, despite its accessibility. *Lobella sauteri* is the first valid bioluminescent species with DNA barcoding in Japan. In this study, we developed a laboratory culture system for *L. sauteri* and successfully raised multiple generations. The luminosity of the fifth generation, raised with feeding non-luminous plasmodia *F. septica,* suggests that *L. sauteri* has an endogenous bioluminescence system independent of any dietary supplementation of luciferins or luciferases. The biochemistry of bioluminescence in *L. sauteri* involves luciferin, luciferase, Mg^2+^ and ATP. Bioluminescence is observed from the tubercules and increases before ecdysis. This regulation of the light might be explained by localization of red pigment rather than by changes in the amounts of luciferin and luciferase. The structure of the luciferin molecule, the protein sequence of the luciferase gene, and ecological role of the enhancement of bioluminescence before ecdysis remain unresolved, but the laboratory culture system provides an opportunity to answer these questions. Taken together, we propose that *L. sauteri* provides a valuable research opportunity for studying an understudied underground bioluminescence system.

## MATERIALS AND METHODS

### Sample collection

Specimens used in this study were collected from litter by the floating method or by using insect collection tubes. For the former method, litter was dumped in a bucket containing water and was stirred. The floating litter is pressed down with a mesh, of which the mesh size is larger than that of springtails, e.g., 2 cm. Springtails floated up due to their hydrophobic nature and were gently scooped with a brush. This method is also useful for collecting other soil animals. For the latter method, fallen leaves and wood sticks are investigated one by one, and the specimens are sucked up by insect collection tubes. *L. sauteri* were collected from Bugenji, Yokohama, Kanagawa, Japan, and Hino, Tokyo, Japan, in September and November 2022, and identified morphologically and by DNA barcode (Kataoka and Nakamori, 2020; Ohira et al., 2023). The identified specimens were cultured in the laboratory.

### Culture for *L. sauteri*

*L. sauteri* was cultured in a plastic container bedded with plaster (Kataoka and Nakamori, 2020; Ohira et al., 2023). The plaster bed was prepared by mixing powder of plaster, water, and black ink or activated charcoal in the ratio of 10:10:1, respectively. *L. sauteri* was fed *Fuligo septica* plasmodia once a week. The plasmodia were cultured on moisture paper in a plastic container and fed crushed oatmeal, as previously described (Kataoka and Nakamori, 2020; Ohira et al., 2023). The piece of paper with the plasmodia is placed on the parafilm on the plaster bed in the culture container when it is fed to *L. sauteri.* Both *L. sauteri* and the plasmodia are kept at 20-22°C. The plasmodia are not fed in the *L. sauteri* culture container. *Lobella sauteri* individuals are transferred to a new culture container using an insect-collection tube, also known as an aspirator, once a week, before mold could grow and infect them.

For the feeding test, *F. septica* plasmodia, mycelia of a bioluminescent mushroom, *Mycena chlorophos,* or dry yeast powder (Asahi, Japan) were fed. The cultured strain of *M. chlorophos* from Hachijo Island, Tokyo, was kept on the yeast potato dextrose agar plate at 30°C. As controls, we also prepared a no-food treatment. Four individuals of *L. sauteri* were placed in the culture container with feeding items and kept at 22°C. The feeding test was performed with three replicates for each condition. The surviving curves (Kaplan–Meier curves) were analyzed by log-rank test, followed by Bonferroni correction for multiple comparisons using survival and survminer packages of R software (version 4.4.0). The ANOVA and the post-ANOVA Tukey's HSD test were performed using car and multicomp packages of R software.

To determine the duration between molting, 50 individuals were kept in a culture case at 22°C and the exuviae were counted the next day. The ecdysis duration was calculated as the number of individuals divided by the number of exuviae. The average number is calculated from 32 replicates.

### Bioluminescence observation

The bioluminescence was observed from *L. sauteri* upon mechanical stimulation and was filmed for still images and a movie with a digital camera, SONY Alpha 7S III (SONY), with a 50 mm macro lens, SEL50M28 (SONY).

### Biochemical assay for bioluminescence

Crude luciferin and luciferase extracts were prepared on ice during all the steps except as specified. Five or ten of the frozen whole bodies of the specimens were homogenized in a 300 µl luciferin extraction buffer with a plastic pestle. The homogenate was centrifuged at 15,000 ***g*** at 4°C for 2 min. The transparent supernatants were separated using Amicon Ultra 100K (Merck Millipore, MA, USA) to obtain smaller and larger molecular weight fractions, namely luciferin and luciferase fractions, respectively. The remaining larger molecules, including proteins, on the filter cup were washed twice with 500 µl of luciferase extraction buffer and recovered to about 50 µl. The composition of the extraction buffers varied for experiments. All the measurements were performed with four experimental replicates and three biological replicates unless specified.

Luminescent activity was measured with a luminometer LuminoSkan (Thermo Fisher Scientific, MA, USA) with 96-well white plates. The light intensity was measured immediately after mixing 50 µl of luciferase solution and 10 µl of luciferin solution for 500 ms, unless specified. The ANOVA and the post-ANOVA Tukey's HSD tests were performed using car and multicomp packages of R software (version 4.4.0).

The *in vitro* luminescence reaction was tested as follows. The luciferin and luciferase fractions were extracted with 20 mM Tris-HCl, pH 8.0 buffer (nacalai tesque, Kyoto, Japan). The larger molecular fraction containing luciferase was diluted 20 times with 100 mM glycine-NaOH, pH 9.0 buffer (Fujifilm Wako Pure Chemical), and the background luminescence was measured for 100 cycles. Luciferase activity was measured after injecting the smaller molecular fraction containing luciferin for 100 cycles. The luciferin fraction was repeatedly tested as follows: the larger molecular fraction containing luciferase was diluted 20 times with 20 mM Tris-HCl, pH 8.0 buffer, and the background luminescence was measured as described above by LuminoSkan for 100 cycles. Then, 50 µl of the same diluted luciferase was injected and measured for 240 cycles. Next, the smaller molecular fraction containing luciferin was injected twice and measured for 240 cycles each time.

The pH dependency of the luciferase activity was investigated as follows. The luciferin and luciferase were extracted with 20 mM Tris-HCl, pH 8.0. The luciferase was diluted 20 times with 100 mM of either Tris-HCl buffer (nacalai tesque, Japan), glycine-NaOH buffer (nacalai tesque), or sodium phosphate buffer at pH 7.0 to 10.0 (Fujifilm Wako Pure Chemical). Luciferase activity was measured as described above by LuminoSkan for 120 cycles, and the total activity was calculated.

The stability of luciferin and luciferase was tested as follows: luciferin and luciferase were extracted with 20 mM glycine-NaOH, pH 9.0. The luciferin and the diluted luciferase with 100 mM glycine-NaOH, pH 9.0 were incubated at 95°C for 5 min. The luciferin was also incubated with or without Proteinase K (0.2 mg/ml final concentration, QIAGEN, Germany) at 37°C for 30 min. The luciferase extract was treated with Proteinase K (0.25 mg/ml final concentration), sodium dodecyl sulfate (SDS, nacalai tesque) (1% final concentration), 2-mercaptoethanol (1% final concentration, nacalai tesque), or ethanol (1% or 5% final concentration, nacalai tesque) at 37°C for 30 min. The remaining activity was measured as described above by LuminoSkan for 120 cycles, and the total activity was calculated.

To determine the cofactors involved in the bioluminescent reaction in *L. sauteri,* we performed the following experiments. The luciferin and luciferase were extracted with 20 mM glycine-NaOH, pH 9.0. The luciferase activity was measured by mixing luciferin and luciferase in a buffer containing 0.5 mM ATP (Merck Millipore), 2.5 mM EDTA (nacalai tesque), and 5 mM of either MgCl_2_ (nacalai tesque), CaCl_2_ (Fujifilm Wako Pure Chemical, Japan), ZnCl_2_ (Fujifilm Wako Pure Chemical), FeCl_2_ (Fujifilm Wako Pure Chemical), or FeCl_3_ (Fujifilm Wako Pure Chemical). The luciferase activity was measured again by mixing luciferin and luciferase in a buffer containing 0.5 mM ATP (Merck Millipore), 5 mM EDTA, 10 mM MgCl_2_, and either 1 mM of ATP, adenosine monophosphate (AMP, Tokyo Chemical Industry Co., Japan), guanosine triphosphate (GTP, Merck Millipore), or coenzyme A (CoA, Oriental Yeast Co., Japan).

The combinational effect of magnesium ions and ATP is tested as follows. The luciferin and luciferase were extracted with 20 mM glycine-NaOH, pH 9.0. The luciferase activity was measured by mixing luciferin and luciferase fractions in a buffer containing 0.5 mM ATP, 5 mM EDTA, and 10 mM MgCl_2_. The photograph of *in vitro* bioluminescence was taken under the following conditions: the luciferin and luciferase were extracted with 20 mM glycine-NaOH at pH 9.0, containing 5 mM EDTA. The luciferase activity was measured by mixing luciferin and luciferase fractions in a buffer containing 10 mM ATP and 5 mM MgCl_2_.

The relative amount of luciferin and luciferase in the red and white tubercular individuals was compared as follows. The luciferin and luciferase were extracted from white tubercular specimens or the specimens molted on that day with 20 mM glycine-NaOH, pH 9.0, containing 5 mM EDTA. The light production was measured by mixing luciferin and luciferase fractions for all the combinations in a buffer containing 1.0 mM ATP, 5 mM EDTA, and 10 mM MgCl_2_. Luciferase activity was measured as described above by LuminoSkan for 120 cycles, and the total activity was calculated. Student's *t*-test was performed using stats packages of R.

### Cross-reactivity with other organisms

To evaluate cross-reactivity with different organisms, luminescence assays were conducted using luciferase and luciferin solutions prepared from various sources. The 10 specimens of *L. sauteri* (approximately 6.2 mg)*,* 50 mg of lyophilized fruiting body of *M. chlorophos*, and 50 mg of plasmodium *F. septica* were homogenized in the extraction buffer (20 mM Glycine-NaOH, 5 mM EDTA, pH 9.0)*.* The crude luciferin and luciferase solutions were separated by ultracentrifugation of homogenates as described above. The extracts of luciferin and luciferase were diluted to 500 µl with the extraction buffer. For firefly luciferase, a purified recombinant luciferase LpLuc1 from *Luciola parvula,* prepared as described before (Bessho-Uehara and Oba, 2017), was prepared in the extraction buffer with a concentration of 1.77 µg/ml (0.45 µg/ml at the measurement). Authentic firefly D-luciferin (Tokyo Chemical Industry) was prepared as 200 nM in the extraction buffer (50 nM at the measurement). The light intensity was measured immediately after mixing 20 µl of luciferase solution, 40 µl of reaction solution (20 mM Glycine-NaOH, 5 mM EDTA, 20 mM MgCl_2_, 20 mM ATP), and 20 µl of luciferin solution with LuminoSkan for 500 ms, 20 cycles, and the total activity was calculated.

### Measurement for the white area on the tubercle

The color change of the tubercle on *L. sauteri* was quantified by analyzing photographs. The individual specimens were kept separately in the culture container and photographed with a digital camera D7500 (Nikon, Japan) every day under the same lighting conditions using a ring light: exposure time 1/200 s, ISO 1000, F/4.8. The image was processed with ImageJ Fiji after the background was trimmed (Fig. S2). The image was split into three color channels (red, blue, and green) by ‘split channel’. The area of the blue channel was calculated as the white area, and the area of the red channel was calculated as the total body area. The white:red ratio was calculated as the white area divided by the red area for individuals every day.

### Measurement of bioluminescent capacity of frozen individuals

To measure total bioluminescent capacity, frozen individuals on a 96-well plate were placed in the photometer, and the light emission was measured for 20 msec for 200 cycles. The total activity was then calculated. Student's *t*-test was performed using stats packages of R.

### Absorbance spectra measurement

The red pigment was extracted from 10 individuals of *L. sauteri.* The sample was homogenized in 200 µl of 20 mM glycine-NaOH buffer at pH 9.0 containing 5 mM EDTA and centrifuged at 15,000 ***g*** for 2 min at 4°C. The red pellet was resuspended in 200 µl of water and mixed with 200 µl of ethyl acetate. After centrifugation at 15,000 ***g*** for 2 min at 4°C, the colored organic layer was collected into a quartz cuvette and the absorbance spectrum was measured using a SpectraMax M5e (Molecular Devices, CA, USA).

### RNA sequencing and transcriptome analysis

Total RNA was extracted using TRIzol reagent (Thermo Fisher Scientific) and treated with DNase (QIAGEN) on column. The purified total RNA was sent to a sequencing service for mRNA-seq transcriptome analysis (Macrogen, Korea). In brief, the concentration and integrity of RNA were quantified by microgel electrophoresis with 2200 TapeStation (Agilent, CA, USA). The mRNA sequencing library was prepared with the TruSeq stranded mRNA library kit. High-throughput short-read sequencing was performed by the next-generation sequencer NovaSeq (Illumina, CA, USA) for paired-end 151 bp. The raw sequence data are available in the DDBJ database (under the bioproject: accession PRJDB18996). The high-quality reads were filtered from raw reads by using fastp (Chen et al., 2018) and trimmed one base from the 5′ end and two bases from the 3′ ends. The filtered reads were *de novo* assembled by Trinity version 2.9.1 (Grabherr et al., 2011). Gene model and protein model were constructed by Transdecoder (Haas et al., 2013). The predicted protein model was validated with the Benchmarking Universal Single-Copy Orthologs (BUSCO) software version 5.8.2 using datasets of artholopoda_odb12 (Manni et al., 2021).

The genes homologous to the known luciferases were investigated by homology search using the Basic Local Alignment Search Tools (BLAST), BLASTp and tBLASTx, and by the phylogenetic analyses. Homologous genes were aligned by MAFFT version 7.490 (Katoh and Standley, 2013) with default settings in Geneious Prime software (version 2023.2.1) (Biomatters). The phylogenetic relationship of firefly luciferase homologs was inferred as follows. The amino acid sequences of beetle homolog sequences were obtained as previously described and aligned by MAFFT with sequences of *L. sauteri* (Fallon et al., 2018). The maximum likelihood tree was generated by IQ-TREE version 2 using the LG+F+I+G4 model chosen according to BIC criteria, and a consensus tree was generated by resampling 1000 replicates of ultrafast bootstrap analysis (Minh et al., 2020).

## Supplementary Material

10.1242/biolopen.061829_sup1 Supplementary information

## References

[BIO061829C1] Allman, G. J. (1851). On the emission of light by *Anurophorus fimetareus*. *Proc. R. Irish Acad.* 5, 125-126.

[BIO061829C2] An, Y.-J., Kim, S. W. and Lee, W.-M. (2013). The collembola *Lobella sokamensis* juvenile as a new soil quality indicator of heavy metal pollution. *Ecol. Indic.* 27, 56-60. 10.1016/j.ecolind.2012.11.017

[BIO061829C3] Bessho-Uehara, M. and Oba, Y. (2017). Identification and characterization of the Luc2-type luciferase in the Japanese firefly, *Luciola parvula*, involved in a dim luminescence in immobile stages. *Luminescence* 32, 924-931. 10.1002/bio.3273 28295969

[BIO061829C4] Bessho-Uehara, M., Konishi, K. and Oba, Y. (2017). Biochemical characteristics and gene expression profiles of two paralogous luciferases from the Japanese firefly *Pyrocoelia atripennis* (Coleoptera, Lampyridae, Lampyrinae): insight into the evolution of firefly luciferase genes. *Photochem. Photobiol. Sci.* 16, 1301-1310. 10.1039/c7pp00110j 28660982

[BIO061829C5] Bessho-Uehara, M., Yamamoto, N., Shigenobu, S., Mori, H., Kuwata, K. and Oba, Y. (2020). Kleptoprotein bioluminescence: *Parapriacanthus* fish obtain luciferase from ostracod prey. *Sci. Adv.* 6, eaax4942. 10.1126/sciadv.aax4942 31934625 PMC6949039

[BIO061829C6] Bessho-Uehara, M., Mallefet, J. and Haddock, S. H. D. (2024). Glowing sea cucumbers: bioluminescence in the Holothuroidea. In *The World of Sea Cucumbers: Challenges, Advances, and Innovations* (eds. A. Mercier, J. F. Hamel, A. Suhrbier and C. Pearce), pp. 361-375. Elsevier.

[BIO061829C7] Chahartaghi, M., Langel, R., Scheu, S. and Ruess, L. (2005). Feeding guilds in Collembola based on nitrogen stable isotope ratios. *Soil Biol. Biochem.* 37, 1718-1725. 10.1016/j.soilbio.2005.02.006

[BIO061829C8] Chen, S., Zhou, Y., Chen, Y. and Gu, J. (2018). fastp: an ultra-fast all-in-one FASTQ preprocessor. *Bioinformatics* 34, i884-i890. 10.1093/bioinformatics/bty560 30423086 PMC6129281

[BIO061829C9] Claes, J. M., Haddock, S. H. D., Coubris, C. and Mallefet, J. (2024). Systematic distribution of bioluminescence in marine animals: a species-level inventory. *Life* 14, 432. 10.3390/life14040432 38672704 PMC11051050

[BIO061829C10] Coubris, C., Duchatelet, L., Delroisse, J., Bayaert, W. S., Parise, L., Eloy, M. C., Pels, C. and Mallefet, J. (2024). Maintain the light, long-term seasonal monitoring of luminous capabilities in the brittle star *Amphiura filiformis*. *Sci. Rep.* 14, 13238. 10.1038/s41598-024-64010-x 38853171 PMC11163003

[BIO061829C11] Davis, M. P., Sparks, J. S. and Smith, W. L. (2016). Repeated and widespread evolution of bioluminescence in marine fishes. *PLoS ONE* 11, e0155154. 10.1371/journal.pone.0155154 27276229 PMC4898709

[BIO061829C12] Duchatelet, L. and Dupont, S. (2024). Marine eukaryote bioluminescence: a review of species and their functional biology. *Mar. Life Sci. Technol.* 10.1007/s42995-024-00250-0 PMC12102453 40417256

[BIO061829C13] Fallon, T. R., Lower, S. E., Chang, C.-H., Bessho-Uehara, M., Martin, G. J., Bewick, A. J., Behringer, M., Debat, H. J., Wong, I., Day, J. C. et al. (2018). Firefly genomes illuminate parallel origins of bioluminescence in beetles*.* *eLife* 7, e36495. 10.7554/eLife.36495 30324905 PMC6191289

[BIO061829C14] Frank, T. M., Widder, E. A., Latz, M. I. and Case, J. F. (1984). Dietary maintenance of bioluminescence in a deep-sea mysid. *J. Exp. Biol.* 109, 385. 10.1242/jeb.109.1.385

[BIO061829C15] Gimenez, G., Metcalf, P., Paterson, N. G. and Sharpe, M. L. (2016). Mass spectrometry analysis and transcriptome sequencing reveal glowing squid crystal proteins are in the same superfamily as firefly luciferase. *Sci. Rep.* 6, 27638. 10.1038/srep27638 27279452 PMC4899746

[BIO061829C16] Grabherr, M. G., Haas, B. J., Yassour, M., Levin, J. Z., Thompson, D. A., Amit, I., Adiconis, X., Fan, L., Raychowdhury, R., Zeng, Q. et al. (2011). Full-length transcriptome assembly from RNA-Seq data without a reference genome. *Nat. Biotechnol.* 29, 644-652. 10.1038/nbt.1883 21572440 PMC3571712

[BIO061829C17] Haas, B. J., Papanicolaou, A., Yassour, M., Grabherr, M., Blood, P. D., Bowden, J., Couger, M. B., Eccles, D., Li, B., Lieber, M. et al. (2013). De novo transcript sequence reconstruction from RNA-seq using the Trinity platform for reference generation and analysis. *Nat. Protoc.* 8, 1494-1512. 10.1038/nprot.2013.084 23845962 PMC3875132

[BIO061829C18] Haddock, S. H. D., Rivers, T. J. and Robison, B. H. (2001). Can coelenterates make coelenterazine? Dietary requirement for luciferin in cnidarian bioluminescence. *Proc. Natl. Acad. Sci. USA* 98, 11148-11151. 10.1073/pnas.201329798 11572972 PMC58698

[BIO061829C19] Harvey, E. N. (1952). *Bioluminescence*. Academic Press.

[BIO061829C20] Hastings, J. W. (1995). Bioluminescence. In *Cell Physiology Source Book* (ed. N. Sperelakis), pp. 665-681. Elsevier.

[BIO061829C21] Haygood, M. G. (1993). Light organ symbioses in fishes. *Crit. Rev. Microbiol.* 19, 191-216. 10.3109/10408419309113529 8305135

[BIO061829C22] He, J., Li, J., Zhang, R., Dong, Z., Liu, G., Chang, Z., Bi, W., Ruan, Y., Yang, Y., Liu, H. et al. (2024). Multiple origins of bioluminescence in beetles and evolution of luciferase function. *Mol. Biol. Evol.* 41, msad287. 10.1093/molbev/msad287 38174583 PMC10798137

[BIO061829C23] Hopkin, S. P. (1997). *Biology of the Springtails*. London, England: Oxford University Press.

[BIO061829C24] Hoskins, J. L., Janion-Scheepers, C., Chown, S. L. and Duffy, G. A. (2015). Growth and reproduction of laboratory-reared neanurid Collembola using a novel slime mould diet. *Sci. Rep.* 5, 11957. 10.1038/srep11957 26153104 PMC4495557

[BIO061829C25] Isobe, M., Kuse, M., Tani, N., Fujii, T. and Matsuda, T. (2008). Cysteine-390 is the binding site of luminous substance with symplectin, a photoprotein from Okinawan squid, *Symplectoteuthis oualaniensis*. *Proc. Jpn. Acad. Ser. B Phys. Biol. Sci.* 84, 386-392. 10.2183/pjab.84.386 PMC3721202 18997450

[BIO061829C26] Johnson, D. G. and Rosenblatt, R. H. (1988). Mechanisms of light organ occlusion in flashlight fishes, family Anomalopidae (Teleostei: Beryciformes), and the evolution of the group. *Zool. J. Linn. Soc.* 94, 65-96. 10.1111/j.1096-3642.1988.tb00882.x

[BIO061829C27] Johnson, F. H., Sugiyama, N., Shimomura, O., Saiga, Y. and Haneda, Y. (1961). Crystalline luciferin from a luminescent fish, *Parapriacanthus beryciformes**.* *Proc. Natl. Acad. Sci. USA* 47, 486-489. 10.1073/pnas.47.4.486 13790263 PMC221477

[BIO061829C28] Joose, E. N. G. (1966). Some observations on the biology of Anurida maritima (Guérin), (Collembola). *Z. Morphol. Ökol. Tiere* 57, 320-328. 10.1007/BF00407599

[BIO061829C29] Kataoka, M. and Nakamori, T. (2020). Food preferences of Collembola for myxomycete plasmodia and plasmodium responses in the presence of Collembola. *Fungal Ecol.* 47, 100965. 10.1016/j.funeco.2020.100965

[BIO061829C30] Katoh, K. and Standley, D. M. (2013). MAFFT multiple sequence alignment software version 7: improvements in performance and usability. *Mol. Biol. Evol.* 30, 772-780. 10.1093/molbev/mst010 23329690 PMC3603318

[BIO061829C31] Kotlobay, A. A., Sarkisyan, K. S., Mokrushina, Y. A., Marcet-Houben, M., Serebrovskaya, E. O., Markina, N. M., Somermeyer, L. G., Gorokhovatsky, A. Y., Vvedensky, A., Purtov, K. V. et al. (2018). Genetically encodable bioluminescent system from fungi. *Proc. Natl. Acad. Sci. USA* 115, 12728-12732. 10.1073/pnas.1803615115 30478037 PMC6294908

[BIO061829C32] Lau, E. S. and Oakley, T. H. (2021). Multi-level convergence of complex traits and the evolution of bioluminescence. *Biol. Rev. Camb. Philos. Soc.* 96, 673-691. 10.1111/brv.12672 33306257

[BIO061829C33] Lau, E. S., Majerova, M., Hensley, N. M., Mukherjee, A., Vasina, M., Pluskal, D., Damborsky, J., Prokop, Z., Delroisse, J., Bayaert, W.-S. et al. (2025). Functional characterization of luciferase in a brittle star indicates parallel evolution influenced by genomic availability of haloalkane dehalogenase. *Mol. Biol. Evol.* msaf081. 10.1093/molbev/msaf081 40181585 PMC12059646

[BIO061829C34] Lee, J. (1976). Bioluminescence of the Australian glow-worm, *Arachnocampa richardsae* Harrison. *Photochem. Photobiol.* 24, 279-285. 10.1111/j.1751-1097.1976.tb06823.x

[BIO061829C35] Manni, M., Berkeley, M. R., Seppey, M., Simão, F. A. and Zdobnov, E. M. (2021). BUSCO update: novel and streamlined workflows along with broader and deeper phylogenetic coverage for scoring of eukaryotic, prokaryotic, and viral genomes. *Mol. Biol. Evol.* 38, 4647-4654. 10.1093/molbev/msab199 34320186 PMC8476166

[BIO061829C36] Messer, C., Walther, J., Dettner, K. and Schulz, S. (2000). Chemical deterrents in podurid Collembola. *Pedobiologia* 44, 210-220. 10.1078/S0031-4056(04)70041-4

[BIO061829C37] Minh, B. Q., Schmidt, H. A., Chernomor, O., Schrempf, D., Woodhams, M. D., Von Haeseler, A. and Lanfear, R. (2020). Corrigendum to: IQ-TREE 2: new models and efficient methods for phylogenetic inference in the genomic era. *Mol. Biol. Evol.* 37, 2461. 10.1093/molbev/msaa131 32011700 PMC7182206

[BIO061829C38] Misof, B., Liu, S., Meusemann, K., Peters, R. S., Donath, A., Mayer, C., Frandsen, P. B., Ware, J., Flouri, T., Beutel, R. G. et al. (2014). Phylogenomics resolves the timing and pattern of insect evolution. *Science* 346, 763-767. 10.1126/science.1257570 25378627

[BIO061829C39] Nakatsu, T., Ichiyama, S., Hiratake, J., Saldanha, A., Kobashi, N., Sakata, K. and Kato, H. (2006). Structural basis for the spectral difference in luciferase bioluminescence. *Nature* 440, 372-376. 10.1038/nature04542 16541080

[BIO061829C40] Nealson, K. H. and Hastings, J. W. (1979). Bacterial bioluminescence: its control and ecological significance. *Microbiol. Rev.* 43, 496-518. 10.1128/mr.43.4.496-518.1979 396467 PMC281490

[BIO061829C41] Nyholm, S. V. and McFall-Ngai, M. (2004). The winnowing: establishing the squid–vibrio symbiosis. *Nat. Rev. Microbiol.* 2, 632-642. 10.1038/nrmicro957 15263898

[BIO061829C42] Oba, Y. (2022). *Luminous Organisms of the World: Diversity, Ecology, and Biochemistry (in Japanese)*. Nagoya: The University of Nagoya Press.

[BIO061829C43] Oba, Y., Iida, K. and Inouye, S. (2009). Functional conversion of fatty acyl-CoA synthetase to firefly luciferase by site-directed mutagenesis: a key substitution responsible for luminescence activity. *FEBS Lett.* 583, 2004-2008. 10.1016/j.febslet.2009.05.018 19450587

[BIO061829C44] Oba, Y., Branham, M. A. and Fukatsu, T. (2011). The terrestrial bioluminescent animals of Japan. *Zool. Sci.* 28, 771-789. 10.2108/zsj.28.771 22035300

[BIO061829C45] Ohira, A., Nakamori, T., Matsumoto, S., Bessho-Uehara, M., Kato, T. and Oba, Y. (2023). Contribution to the taxonomy of Lobellini (Collembola: Neanurinae) and investigations on luminous Collembola from Japan. *Zootaxa* 5325, 63-89. 10.11646/zootaxa.5325.1.4 38220925

[BIO061829C46] Osozawa, S., Oba, Y., Kwon, H.-Y. and Wakabayashi, J. (2015). Vicariance of *Pyrocoelia* fireflies (Coleoptera: Lampyridae) in the Ryukyu Islands, Japan. *Biol. J. Linn. Soc. Lond.* 116, 412-422. 10.1111/bij.12595

[BIO061829C47] Petushkov, V. N., Dubinnyi, M. A., Tsarkova, A. S., Rodionova, N. S., Baranov, M. S., Kublitski, V. S., Shimomura, O. and Yampolsky, I. V. (2014). A novel type of luciferin from the Siberian luminous earthworm *Fridericia heliota*: structure elucidation by spectral studies and total synthesis. *Angew. Chem. Int. Ed Engl.* 53, 5566-5568. 10.1002/anie.201400529 24737705

[BIO061829C48] Potapov, A. A., Semenina, E. E., Korotkevich, A. Y., Kuznetsova, N. A. and Tiunov, A. V. (2016). Connecting taxonomy and ecology: trophic niches of collembolans as related to taxonomic identity and life forms. *Soil Biol. Biochem.* 101, 20-31. 10.1016/j.soilbio.2016.07.002

[BIO061829C49] Powell, G. S., Saxton, N. A., Pacheco, Y. M., Stanger-Hall, K. F., Martin, G. J., Kusy, D., Felipe Lima Da Silveira, L., Bocak, L., Branham, M. A. and Bybee, S. M. (2022). Beetle bioluminescence outshines extant aerial predators. *Proc. Biol. Sci.* 289, 20220821. 10.1098/rspb.2022.0821 35855602 PMC9297012

[BIO061829C50] Robinson, J. D., Flashner, M. S. and Marin, G. K. (1978). Inhibition of the (Na^+^+K^+^)-dependent ATPase by inorganic phosphate. *Biochim. Biophys. Acta Biomembr.* 509, 419-428. 10.1016/0005-2736(78)90236-5 148911

[BIO061829C51] Sano, T., Kobayashi, Y., Sakai, I., Ogoh, K. and Suzuki, H. (2019). Ecological and histological notes on the luminous springtail, Lobella sp. (Collembola: Neanuridae), discovered in Tokyo, Japan. In *Bioluminescence - Analytical Applications and Basic Biology* (ed. J. Thirumalai). 10.5772/INTECHOPEN.88321

[BIO061829C52] Schnitzler, C. E., Pang, K., Powers, M. L., Reitzel, A. M., Ryan, J. F., Simmons, D., Tada, T., Park, M., Gupta, J., Brooks, S. Y. et al. (2012). Genomic organization, evolution, and expression of photoprotein and opsin genes in *Mnemiopsis leidyi*: a new view of ctenophore photocytes. *BMC Biol.* 10, 107. 10.1186/1741-7007-10-107 23259493 PMC3570280

[BIO061829C53] Schramm, S. and Weiß, D. (2024). Bioluminescence - the vibrant glow of nature and its chemical mechanisms. *Chembiochem* 25, e202400106. 10.1002/cbic.202400106 38469601

[BIO061829C54] Shimomura, O. (1981). A new type of ATP–activated bioluminescent system in the millipede *Luminodesmus sequoiae*. *FEBS Lett.* 128, 242-244. 10.1016/0014-5793(81)80090-7

[BIO061829C55] Shimomura, O. and Yampolsky, I. V. (2019). *Bioluminescence: Chemical Principles and Methods*, 3rd edn. World Scientific.

[BIO061829C56] Tanet, L., Martini, S., Casalot, L. and Tamburini, C. (2020). Reviews and syntheses: bacterial bioluminescence – ecology and impact in the biological carbon pump. *Biogeosciences* 17, 3757-3778. 10.5194/bg-17-3757-2020

[BIO061829C57] Trimmer, B. A., Aprille, J. R., Dudzinski, D. M., Lagace, C. J., Lewis, S. M., Michel, T., Qazi, S. and Zayas, R. M. (2001). Nitric oxide and the control of firefly flashing. *Science* 292, 2486-2488. 10.1126/science.1059833 11431567

[BIO061829C58] Tsuji, F. I. (1985). ATP-dependent bioluminescence in the firefly squid, *Watasenia scintillans*. *Proc. Natl. Acad. Sci. USA* 82, 4629-4632. 10.1073/pnas.82.14.4629 16593580 PMC390439

[BIO061829C59] Tsuji, F. I. and Leisman, G. B. (1981). K+/Na+-triggered bioluminescence in the oceanic squid *Symplectoteuthis oualaniensis*. *Proc. Natl. Acad. Sci. USA* 78, 6719-6723. 10.1073/pnas.78.11.6719 16593119 PMC349121

[BIO061829C60] Viviani, V. R., Okawachi, F. M., Scorsato, V. and Abdalla, F. C. (2008). CCD imaging of basal bioluminescence in larval fireflies: clues on the anatomic origin and evolution of bioluminescence. *Photochem. Photobiol. Sci.* 7, 448-452. 10.1039/b718016k 18385887

[BIO061829C61] Warner, J. A. and Case, J. F. (1980). The zoogeography and dietary induction of bioluminescence in the midshipman fish, *Porichthys notatus*. *Biol. Bull.* 159, 231-246. 10.2307/1541021

[BIO061829C62] Watkins, O. C., Sharpe, M. L., Perry, N. B. and Krause, K. L. (2018). New Zealand glowworm (*Arachnocampa luminosa*) bioluminescence is produced by a firefly-like luciferase but an entirely new luciferin. *Sci. Rep.* 8, 3278. 10.1038/s41598-018-21298-w 29459729 PMC5818473

[BIO061829C63] Webster, J. J., Chang, J. C., Manley, E. R., Spivey, H. O. and Leach, F. R. (1980). Buffer effects on ATP analysis by firefly luciferase. *Anal. Biochem.* 106, 7-11. 10.1016/0003-2697(80)90111-6 7191217

[BIO061829C64] Yoshida, M.-A., Imoto, J., Kawai, Y., Funahashi, S., Minei, R., Akizuki, Y., Ogura, A., Nakabayashi, K., Yura, K. and Ikeo, K. (2020). Genomic and Transcriptomic Analyses of Bioluminescence Genes in the Enope Squid *Watasenia scintillans*. *Mar. Biotechnol.* 22, 760-771. 10.1007/s10126-020-10001-8 PMC7708342 33098466

