# Supplementary material for: Biochemical basis of endogenous bioluminescent springtail Lobella sauteri (Collembola)
Source: Biol Open. 2025 May 12;14(5):bio061829. doi: 10.1242/bio.061829 (PMC12091230; doi:10.1242/bio.061829)
Supplement: Supplementary information [file biolopen-14-061829-s1.pdf]

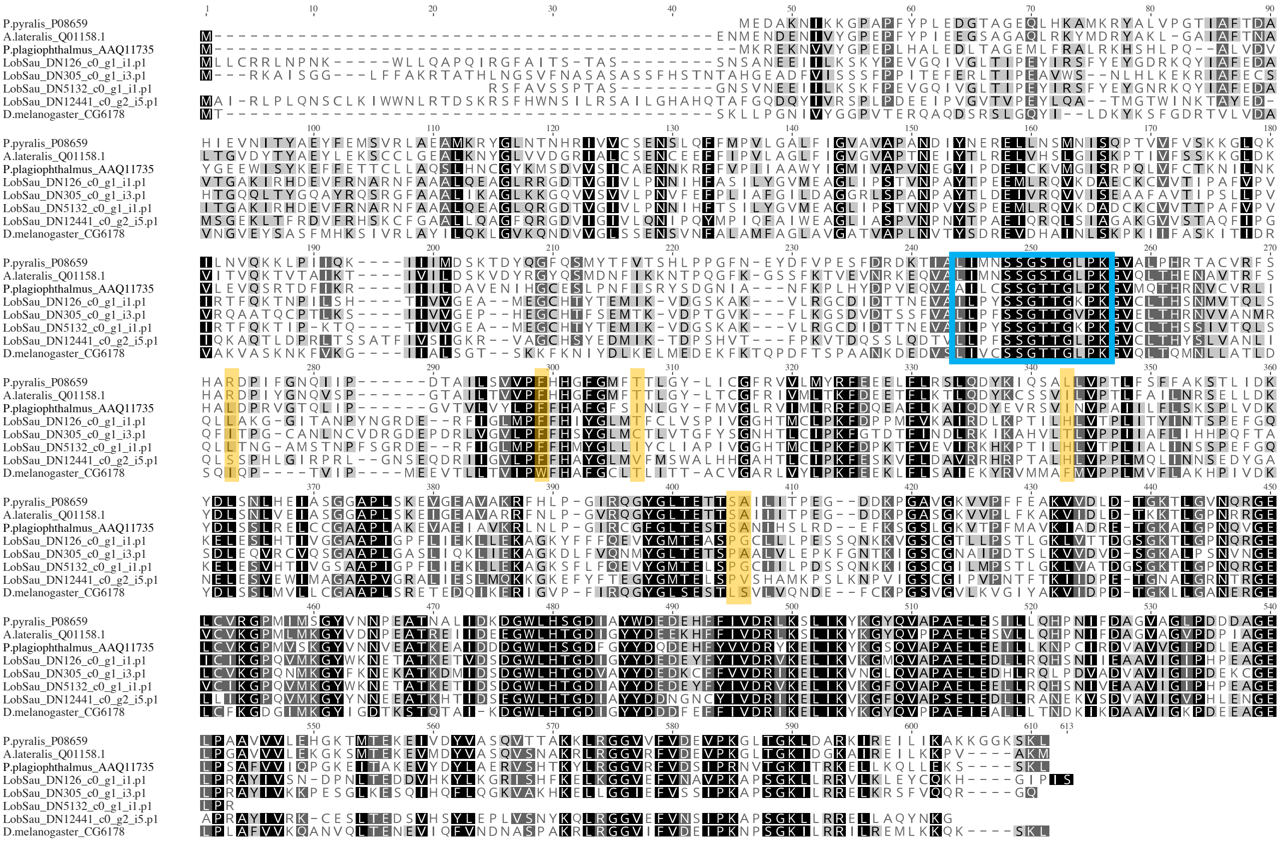

Fig. S1. Protein alignment of firefly luciferase homologs.

Four predicted proteins in *L. sauteri* homologous to firefly luciferase were aligned with the firefly luciferases, the click beetle luciferase, and non-luciferase homolog in *Drosophila melanogaster* GC76178. AMP-binding site is indicated as open blue square. Seven key amino acid residues bound to luciferin are indicated as orange filled square.

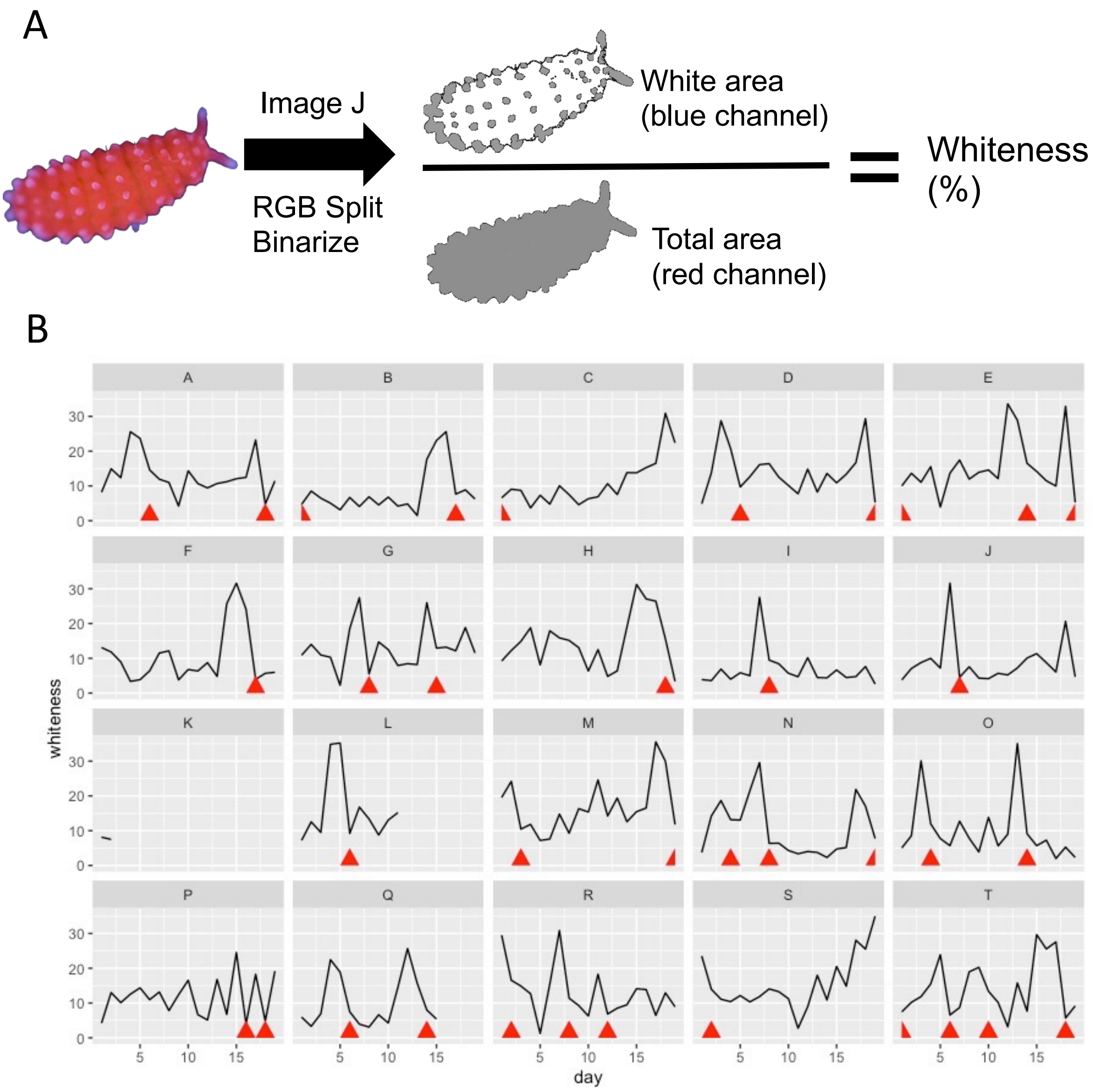

Fig. S2. Changes of whiteness in whole body.

(A) Schematic image of how to determine the whiteness. Individuals were photographed everyday and determined whiteness by calculating white area divided by total area.

(B) Changes of whiteness of 20 individuals of *L. sauteri.* The day exuviate was found, suggesting ecdysis was happened with in 24 hours, is indicated with red triangle.

Table S1. Number of eggs in masses and hatching duration of *Lobella sauteri*

Mass ID	Number of eggs	Hatched	Ratio (%)	Days until hatch	Average
1	11	8	73	14-18	14.6
2	9	9	100	15-21	17.7
3	10	9	90	15-19	14.2

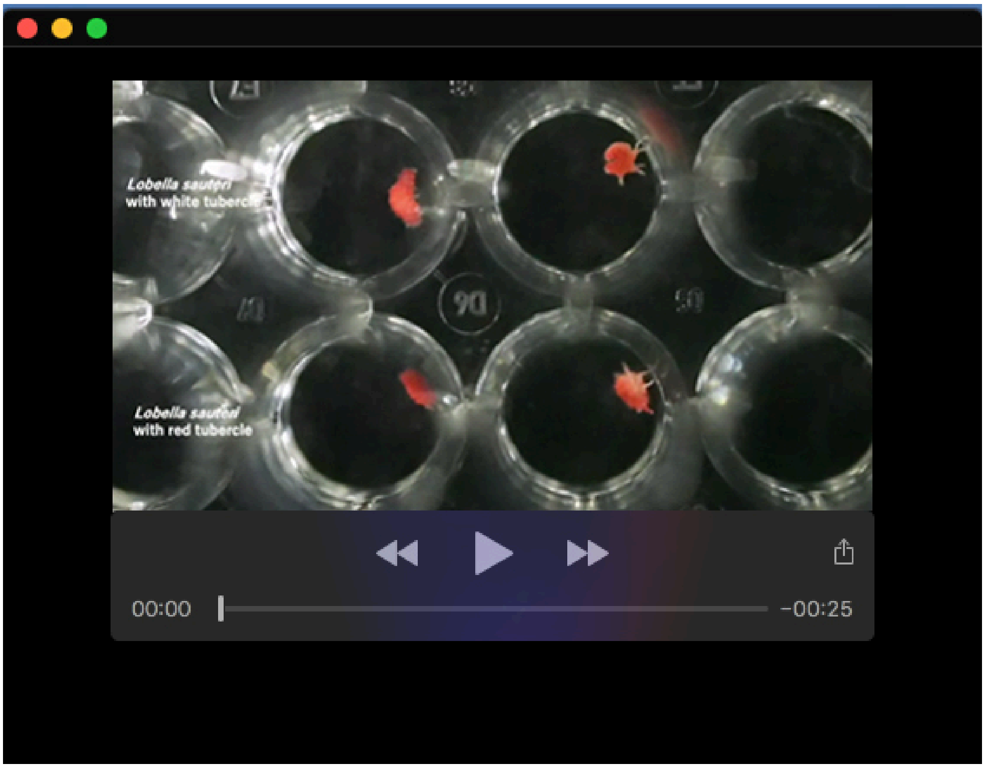

Movie 1. Bioluminescence of *Lobella sauteri* with white or red tubercles

Live specimens were placed in wells, and air was blown over them to induce bioluminescence. The specimens with white tubercles (the two individuals at the top row) emitted brighter green light compared to those with red tubercles (the two individuals at the bottom row).
